# The Mycorrhizal Fungal Species *Funneliformis mosseae* Reshapes Rhizosphere Microbial Metabolic Pathways of Soybeans Under Tetracycline Stress

**DOI:** 10.1002/mbo3.70385

**Published:** 2026-08-30

**Authors:** Chao Yang, Baiyan Cai, Harry Lye Hin Chong, Juan Li, Linghui Li, Jean Wan Hong Yong, Khim Phin Chong

**Affiliations:** ^1^ Department of Science Heihe University Heihe China; ^2^ Biophysics Research Group, Faculty of Science and Technology Universiti Malaysia Sabah Jalan UMS Kota Kinabalu Sabah Malaysia; ^3^ Engineering Research Center of Agricultural Microbiology Technology, College of Heilongjiang Province & School of Life Sciences Heilongjiang University Harbin China; ^4^ Department of Biosystems and Technology Swedish University of Agricultural Sciences Alnarp Sweden; ^5^ Brightlands Future of Farming Institute, Faculty of Science and Engineering Maastricht University Venlo Netherlands

**Keywords:** arbuscular mycorrhizal fungi, correlation network analysis, microbial interactions, rhizosphere metabolome, soil metabolomics

## Abstract

Studies have demonstrated that arbuscular mycorrhizal fungi (AMF) can alter the rhizosphere microbial communities during exposure to tetracycline, thereby alleviating the adverse effects of antibiotics. We hypothesized that *Funneliformis mosseae* (*F. mosseae*) reshapes soil metabolite composition and associated‐microbial pathways under tetracycline stress and that these metabolite changes are associated with the rhizosphere microbial communities. To test this hypothesis, microbiological profiling with metabolomics approaches were carried out to investigate how AMF reshape rhizosphere metabolite profiles in soybeans undergoing tetracycline stress. Specifically, inoculation with *F. mosseae* altered the soil metabolite profiles, with 34 differential metabolites identified, including 14 upregulated and 20 downregulated compounds, mainly amino acids, sugar phosphates, fatty acids, polyphenols, triterpenes, and heterocyclic compounds. In addition, 19 differential metabolic pathways were identified and classified into four metabolic clusters: polyunsaturated fatty acids and oxidative stress‐related metabolism, carbon source and energy metabolism, amino acid and sulfur‐containing metabolism, and secondary metabolism and defense responses. The correlation analysis between soil metabolites and published rhizosphere microbial data revealed 14 metabolites, including Trehalose‐6‐phosphate, increased with bacterial abundance, while five metabolites decreased; similarly, four metabolites increased and three decreased with fungal abundance. Correlation network analysis further indicated that 16 differential metabolites were significantly associated with 8 differential bacterial genera, and 12 differential metabolites were significantly associated with six fungal genera. Overall, our findings demonstrated that *F. mosseae* plays a crucial role in regulating microbial metabolism and interactions in tetracycline‐contaminated soils, providing valuable insights into the mechanisms by which AMF alleviate tetracycline‐induced stress in soils.

## Graphical abstract

The presence of residual antibiotics (tetracycline) poses a threat to food safety in the black soils of northeastern China and a mycorrhizal fungal species *Funneliformis mosseae* could mitigate the negative effects. This study tracked the changes in soybeans' rhizospheric microbial profiles and the associated metabolites during the bioremediation process.

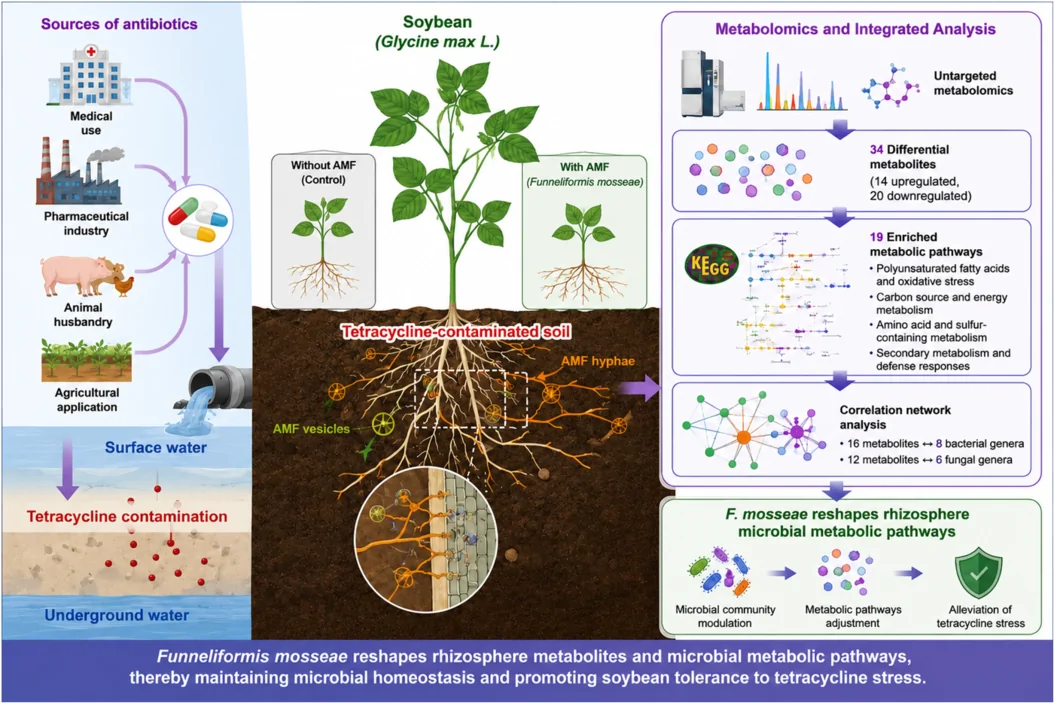

## 1 Introduction

Antibiotics are widely used in livestock farming in rural China due to their low cost, high efficiency and broad‐spectrum antimicrobial properties (Li et al. 2015). China is the world's largest consumer of tetracycline, with the majority being applied in the livestock industry (Han et al. 2021; Yang et al. 2025). Tetracycline is a broad‐spectrum antibiotic belonging to the tetracycline class and is extensively used in veterinary medicine and livestock production for disease prevention and growth promotion (Li et al. 2025). However, many antibiotics cannot be completely absorbed or metabolized in the animal gut, and approximately 40%–90% are excreted into the environment in their original form (Lin et al. 2021; QIN et al. 2022). Due to its high stability, strong adsorption to soil particles and limited biodegradability, tetracycline can persist in agricultural soils for extended periods after being introduced through livestock manure and wastewater, making it one of the most frequently detected antibiotic contaminants in terrestrial ecosystems (Wang et al. 2024). These residual antibiotics not only pose a threat to human health but also interfere with the growth and metabolic activities of plants, animals and soil microorganisms (Han et al. 2019; Zakordonets et al. 2016). Some previous studies shown that excessive accumulation of tetracycline in soil disrupts soil ecological balance by suppressing microbial activity, impairing plant nitrogen fixation and reducing the availability of essential trace elements, ultimately leading to soil fertility degradation (Cycoń et al. 2019; Semedo et al. 2018). Therefore, tetracycline, as an emerging environmental pollutant, has attracted increasing attention from the scientific community.

It is well known that arbuscular mycorrhizal fungi (AMF) play a vital role in enhancing plant resilience to biotic and abiotic stresses, such as heavy metal contamination (Hu et al. 2021), drought stress (Ren et al. 2019) and organic pollutants (Gao et al. 2010). Studies shown AMF can enhance plant antioxidant enzyme activities, increase the abundance of soil microbial communities and improve soil enzyme activities (Yang et al. 2019). Furthermore, Wang et al. (2020) reported that AMF have a positive effect in reducing organic pollutant residues in plants. The underlying mechanisms may include promoting nutrient uptake, increasing stress‐related enzyme activities, optimizing the structure of microbial communities in contaminated soils and enhancing microbial utilization, thereby facilitating the remediation of polluted soils. Among AMF, *Funneliformis mosseae* (*F. mosseae*) is a common indigenous species widely distributed in the Mollisol region of Northeast China and exhibits high colonization efficiency and adaptability to local soil environments. Therefore, *F. mosseae* was selected as the experimental AMF species in the present study.

Soil microbial communities serve as the central engine driving various energy‐intensive chemical transformation processes in soils. They consume and synthesize diverse chemical substrates during metabolic activities and generate a wide range of metabolites as byproducts (Honeker et al. 2021). Therefore, the activity of soil microorganisms is inextricably linked to soil metabolites. It has been reported that antibiotics can alter soil microbial diversity and affect community functions (Lin et al. 2016), including key ecological processes such as nitrogen and carbon cycling (Wepking et al. 2019). In recent years, the emergence of soil metabolomics has provided novel tools for identifying biochemical intermediates in interacting metabolic pathways, thereby deepening our systematic understanding of soil biological processes (Withers et al. 2020).

The diversity of soil metabolites plays a crucial role in reflecting soil functions and quality (Seifert et al. 2016) and also serves as an important carbon source for soil microorganisms (Swenson et al. 2018). Moreover, specific soil metabolites can regulate the structure and activity of soil microbial communities, while changes in microbial communities can, in turn, reshape the composition of metabolites (Liang et al. 2017; Lucas et al. 2016). Thus, soil metabolomics not only provides a powerful tool for studying soil microorganisms but also serves as an effective approach to elucidate the impacts of exogenous disturbances on biological systems (Wu et al. 2022). In addition, soil metabolomics can be used to explore changes in soil metabolites and metabolic pathways, and when integrated with microbiome data, it helps identify potential bioindicators and enables coupled analyses between compounds and soil microbial communities (Song et al. 2020). Combining microbial community composition with the profiles of soil metabolites and functional metabolic pathways offers deeper insights into the complex biological processes occurring in soils (Morton et al. 2019).

In our previous study (Yang et al. 2025), we demonstrated that *F. mosseae* significantly altered the abundance and community composition of rhizosphere microorganisms in soybean under tetracycline stress. However, although changes in microbial community structure were clearly observed, the underlying metabolic mechanisms driving these changes remained unclear. In particular, it is still unknown whether AMF‐mediated alterations in rhizosphere metabolites contribute to the restructuring of microbial communities and their metabolic functions. Therefore, the present study extends our previous work by integrating soil metabolomics with previously obtained rhizosphere microbial community data to investigate the relationships between differential metabolites and microbial taxa under tetracycline stress. This integrated approach provides mechanistic insights into how *F. mosseae* regulates rhizosphere microbial metabolism and interactions, thereby providing scientific evidence for the application of AMF in the remediation of antibiotic‐contaminated agricultural soils and the development of sustainable soil management strategies.

We hypotheses that (1) Under tetracycline stress, *F. mosseae* can reshape the composition and abundance of soil metabolites in the soybean rhizosphere and influence the metabolic pathways of soil microorganisms. (2) The differentially expressed metabolites identified are likely to be closely associated with the activity and community structure of soil microorganisms.

## 2 Materials and Methods

### 2.1 Experimental Materials

The experiment was conducted at the experimental field of Harbin Institute of Technology, China (longitude 125°42′–130°10′ E, latitude 44°04′–46°40′ N). The parent material of the experimental plots was cold‐region black calcareous soil, and its initial physicochemical properties are shown in Table S1. The topsoil layer (0–20 cm) was collected, air‐dried, passed through a 1 cm sieve to remove debris and large particles and subsequently filled into pots with a height of 20 cm and a diameter of 16 cm for later use. The host plant used was the widely cultivated soybean (*Glycine max* (L.) Merr.) variety HN48 (high‐protein type) in Harbin and surrounding areas of Heilongjiang Province. The nutritional composition of soybean seeds is provided in Table S2. Arbuscular mycorrhizal fungus (AMF) inoculum was *F. mosseae*, originally obtained from the Institute of Plant Nutrition and Resources, Beijing Academy of Agriculture and Forestry Sciences. The AMF inoculum was propagated in‐house using alfalfa (*Medicago sativa* L.) as the host plant in a sterilized substrate consisting of soil, sand and vermiculite. The inoculum was collected only after the root colonization rate of alfalfa exceeded 90%, indicating a high infectivity potential. The harvested inoculum, including colonized root fragments and the surrounding growth substrate, was homogenized and stored at −20°C until use. The inoculum contained 21–26 spores g^−1^. Tetracycline hydrochloride (C_22_H_24_N_2_O_8_·HCl; CAS 64–75‐5; analytical grade, purity ≥ 99%) was purchased from Sumed Biotech Co. Ltd., and used to establish tetracycline stress conditions.

### 2.2 Experimental Design

The experiment was conducted using a pot setup, with each pot filled with 4 kg of air‐dried soil. Tetracycline was applied at a concentration of 40 mg/kg, and *F. mosseae* was inoculated at 10 g/kg of soil(Shi et al. 2025; Yang et al. 2025). The experiment was arranged as a completely randomized design consisting of four treatments: CK (natural growth control), ST (tetracycline only), SF (*F. mosseae* only), and FT (tetracycline + *F. mosseae*), each with three biological replicates. Detailed treatment grouping is provided in Table S3.

Initially, five soybean seedlings were sown per pot (ensuring more than three seedlings germinated per pot), and only three seedlings were retained after germination for continued growth. The experiment commenced from May 22 to August 28, 2023, with 15 pots per treatment, totaling 60 pots. During soybean growth, irrigation and weeding were performed as required. Pots were arranged randomly, and their positions were regularly changed to minimize edge effects on the experimental outcomes.

### 2.3 Soil Sample Collection

Soil samples were collected every 15 days starting from 30 days after soybean planting, corresponding to the seedling stage (30 days), flower bud differentiation stage (45 days), flowering and pod‐setting stage (60 days), pod‐filling stage (75 days), and maturity stage (90 days). At each sampling time, three pots were randomly selected, comprising a total of nine soybean plants (three plants per pot). Although samples were collected throughout the entire experimental period to monitor soybean growth and rhizosphere changes, only the rhizosphere soil samples collected at 75 days were used for the metabolomic analysis in the present study. Based on our previous study (Yang et al. 2025), 75 days after planting corresponded to the peak colonization of *F. mosseae* and the highest rate of tetracycline degradation in the rhizosphere. Therefore, this time point was selected as the representative stage to investigate the effects of *F. mosseae* on rhizosphere soil metabolism under tetracycline stress. During sampling, surface debris was first removed from each pot, and the entire plants were carefully uprooted. Rhizosphere soil approximately 5 mm from the root surface was gently brushed off using a sterilized brush, depending on soil adhesion. The collected soil was passed through a 2 mm sieve, rapidly frozen in liquid nitrogen for 1 min, and then stored at −80°C until metabolomic analysis.

### 2.4 Soil Metabolomics Analysis

Metabolites Extraction: A 50 mg aliquot of the soil sample was transferred into an Eppendorf tube. Then, 200 μL of extraction solution (a 1:1 mixture of acetonitrile and methanol) containing a mixture of isotopically labelled internal standards was added. These internal standards include labelled amino acids, organic acids, sugars, lipids, and nucleosides. The mixture was vortexed for 30 s, sonicated in an ice‐water bath for 10 min and then incubated at −40°C for 1 h to precipitate proteins. Then, the sample was centrifuged at 13800 × g for 15 min at 4°C. The resulting supernatant was transferred to a fresh glass vial for analysis. In this study, the quality control (QC) sample was prepared by pooling equal volumes of supernatants from all experimental samples. Three biological replicates were included for each treatment and QC samples were analyzed at regular intervals throughout the UHPLC‐MS/MS sequence to monitor instrument stability and analytical reproducibility.

The UHPLC‐MS/MS Analysis: A Vanquish (Thermo Fisher Scientific) ultra‐high performance liquid chromatograph was used to perform chromatographic separation of the target compounds using the Waters ACQUITY UHPLC BEH Amide liquid chromatography column. Phase A of liquid chromatography was an aqueous phase and phase B was acetonitrile. Sample tray temperature: 4°C, injection volume: 2 μL. The Orbitrap Exploris 120 mass spectrometer is capable of collecting primary and secondary mass spectrometry data under the control software (Xcalibur, version 4.4, Thermo). The detailed parameters were as follows: Sheath gas flow rate: 50 Arb, Aux gas flow rate: 15 Arb, Capillary temperature: 320°C, Spray Voltage: 3.8 kV (positive) or −3.4 kV (negative).

Data preprocessing and annotation: The raw data were converted to the mzXML format using ProteoWizard and processed with an in‐house programme, which was developed using R and based on XCMS, for peak detection, extraction, alignment and integration. Then an in‐house MS2 database was applied in metabolite annotation.

### 2.5 Statistical Analysis

The raw UHPLC‐MS/MS data were preprocessed through a series of steps, including peak filtering, data normalization and missing value imputation. Principal component analysis (PCA) and orthogonal partial least squares discriminant analysis (OPLS‐DA) were performed using R software (version 4.3.3) with the ggplot2 package (3.3.2) to identify and validate differences in metabolites among treatment groups. Volcano plots were employed to visualize differentially expressed metabolites. Hierarchical clustering and metabolic pathway enrichment analyses were conducted using the gplots package (3.0.4) in R, enabling visualization of differences in rhizosphere microbial metabolite composition across treatments. Differential metabolites and pathways were further mapped and constructed based on fold change and p‐values using open‐source Python libraries (version 3.13.5). Two‐way analysis of variance (ANOVA) was performed in SPSS 26.0, followed by Tukey's HSD post hoc test to assess the statistical significance of differences in relative metabolite abundances.

### 2.6 Integrated Analysis of Soil Microorganisms and Metabolites

We integrated identified differential bacterial and fungal genera in the rhizosphere from our previous study (Yang et al. 2025) to the current study. For each biological sample, the relative abundances of all differential bacterial or fungal genera were summed to obtain the summed relative abundance of differential microbial taxa. Pearson or Spearman correlation coefficients and corresponding *p*‐values were then calculated to evaluate the relationships between the summed relative abundance of differential microbial taxa and differential metabolites. Metabolites with |r | ≥ 0.6 and *p* ≤ 0.05 were considered significantly correlated and visualized using scatter plots. Furthermore, a genus‐level co‐occurrence network was constructed to investigate associations between differential metabolites and individual bacterial or fungal genera. Significant correlations were defined as |r | ≥ 0.8 and *p* ≤ 0.05 and visualized using the R package *igraph* (version 1.2.5).

## 3 Result

### 3.1 Soil Metabolomic Data Analysis and Classification Annotation

**Figure 1:**
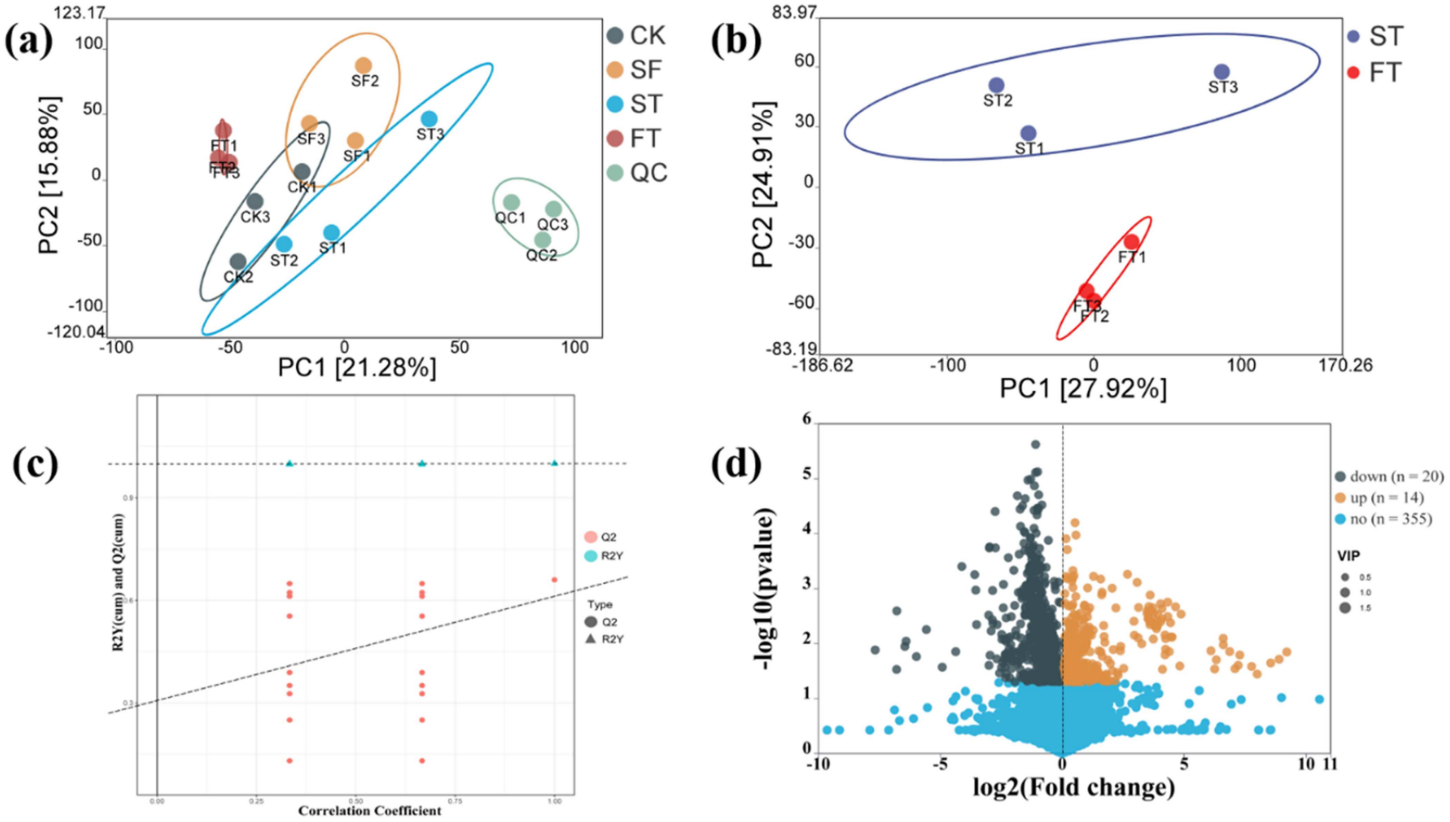
Effects of *F. mosseae* colonization on the soybean rhizosphere soil metabolome under tetracycline stress. (a) Principal component analysis (PCA) of rhizosphere soil metabolites; (b) Unsupervised PCA comparing FT and ST groups; (c) Cross‐validation of the PLS‐DA model; (d) Volcano plot highlighting differential metabolites between FT and ST groups. The confidence intervals in (a) and (b) are set at 85%. CK: soil with soybean only; SF: soil with soybean and *F. mosseae*; ST: soil with soybean and tetracycline; FT: soil with soybean, *F. mosseae*, and tetracycline; QC: quality control samples. QC samples were used to evaluate the stability of the analytical instrument; smaller variations indicate higher methodological stability and better data quality.

A total of 389 known metabolites were identified across all soil samples. After the introduction of quality control samples, principal component analysis (PCA) was performed on the four treatment groups. The PCA score plot showed clustering of samples within each treatment group, while separation was observed among different treatment groups (Figure 1a). The high overlap of the TIC chromatograms among QC samples (Figure S1) indicated good analytical reproducibility and instrument stability. PC1 and PC2 together explained 37.16% of the total variation. To further determine whether the colonization of *F. mosseae* under tetracycline stress affected the metabolic composition between treatment and control groups, unsupervised PCA was first applied to compare the groups (Figure 1b). The results revealed a difference in metabolite composition between the FT and ST treatment groups, with PC1 and PC2 explaining 27.92% and 24.91% of the variation, respectively. To evaluate the reliability of the OPLS‐DA model, permutation testing was conducted. The results showed that the model exhibited a good fit (R^2^Y = 0.95, Q^2^Y = –0.88) (Figure 1c), indicating that it was suitable for the screening of differential metabolites. Subsequently, volcano plots were used to identify differential metabolites between the *F. mosseae*‐colonized treatment group and the control group (Figure 1d). The results demonstrated that *F. mosseae* colonization significantly altered the expression of rhizosphere soil metabolites under tetracycline stress.

To further investigate the biological functionality of rhizosphere soil metabolites and the probable metabolic pathways in which they are involved under tetracycline stress, the detected and identified soil metabolites were annotated against the KEGG database. Pathway annotation of the metabolites from the *F. mosseae* treatment was performed using the KEGG Pathway database (Figure S2). The results showed that, in the comparison group (FT‐vs‐ST), a total of 60 metabolites were assigned to six major KEGG metabolic pathway categories. Specifically, 19 metabolites were classified under Organismal Systems, 29 under Metabolism, 3 under Cellular Processes, 4 under Human Diseases, 4 under Environmental Information Processing, and 1 under Genetic Information Processing. These metabolites were further divided into secondary pathway categories, and the number of metabolites annotated to each category is shown in Figure S2, such as Endocrine system, Lipid metabolism, and Amino acid metabolism. Overall, the results indicated that the metabolites were predominantly involved in pathways related to Metabolism and Organismal Systems.

### 3.2 Analysis of Differential Soil Metabolites

**Figure 2:**
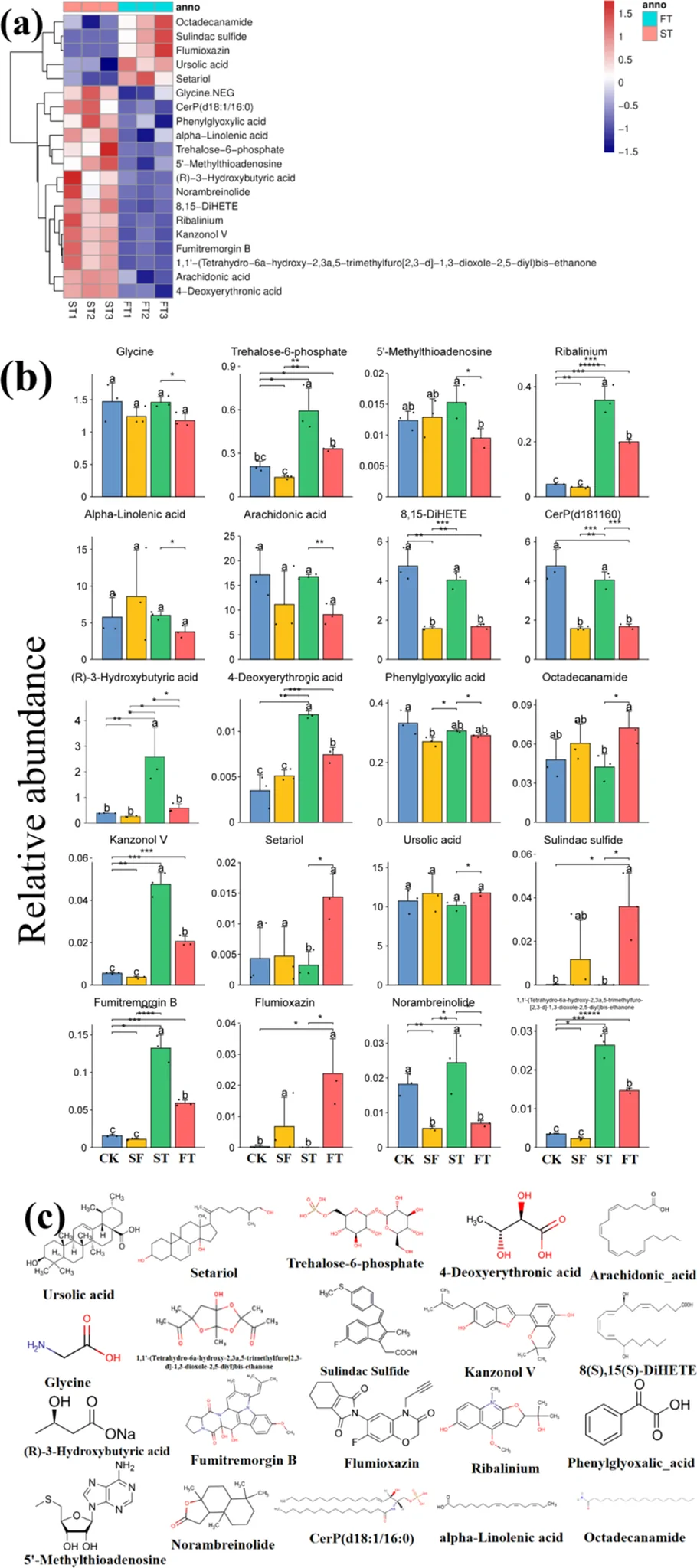
Analysis of differential metabolites in the rhizosphere soil of the FT‐vs‐ST comparison group. (a) Heatmap of the top 20 differential metabolites in the soybean rhizosphere for the FT‐vs‐ST comparison group, (b) Bar plots showing the relative abundance of these 20 differential metabolites across all treatments, (c) Chemical structures of the 20 differential metabolites. Graphs a, b, and c indicate significant differences (*p* < 0.05), and significance was assessed using the Tukey HSD test. *Indicates the significance of the difference between the two groups, with more asterisks representing greater significance (t‐test). CK: soil with soybean only; SF: soil with soybean and *F. mosseae*; ST: soil with soybean and tetracycline; FT: soil with soybean, *F. mosseae*, and tetracycline.

Since metabolomic analysis is based on pairwise comparisons between two treatment groups, each treatment was subdivided into six comparison groups (FT‐vs‐CK, FT‐vs‐SF, FT‐vs‐ST, SF‐vs‐CK, SF‐vs‐ST and ST‐vs‐CK). We calculated Euclidean distance matrices using the quantitative values of the differential metabolites and performed clustering using the complete linkage method. As a result, 47, 47, 34, 44, 36, and 33 differential metabolites were identified in each comparison, respectively. Among them, 35, 26, 14, 6, 5, and 26 metabolites were upregulated, while 12, 21, 20, 38, 31, and 7 were downregulated (Figure S3). To further investigate the effect of *F. mosseae* colonization on soil microbial differential metabolites under tetracycline stress, differential metabolites in the FT versus ST group were selected, and both cationic and anionic metabolites were combined. The top 20 differential metabolites are shown in Figure 2a. Comparison of their relative abundance (Figure 2b) revealed that the upregulated metabolites included Octadecanamide, Sulindac sulfide, Flumioxazin, Ursolic acid, and Setariol, which increased by 0.71‐fold, 600‐fold, 484.71‐fold, 0.16‐fold and 3.5‐fold, respectively. The downregulated metabolites included Glycine, CerP(d18:1/16:0), Phenylglyoxylic acid, *α*‐Linolenic acid, Trehalose‐6‐phosphate, 5'‐Methylthioadenosine, (R)‐3‐Hydroxybutyric acid, norambreinolide, 8,15‐DiHETE, ribalinium, kanzonol V, fumitremorgin B, 1,1'‐(tetrahydro‐6a‐hydroxy‐2,3a,5‐trimethylfuro[2,3‐d]‐1,3‐dioxole‐2,5‐diyl)bis‐ethanone, arachidonic acid, and 4‐deoxyerythronic acid, which decreased by 23.73%, 16.46%, 5.30%, 59.53%, 79.02%, 61.05%, 344.06%, 248.57%, 140.35%, 75.54%, 132.20%, 123.69%, 79.59%, 84.30%, and 59.46%, respectively. Their molecular structures are shown in Figure 2c. These differential metabolites include primary metabolites (such as amino acids, sugar phosphates, and fatty acids) and secondary metabolites (such as polyphenols, triterpenes, and heterocyclic compounds), indicating that *F. mosseae* colonization significantly altered the accumulation and composition of soil metabolites.

### 3.3 Analysis of Differential Soil Metabolic Pathways

**Figure 3:**
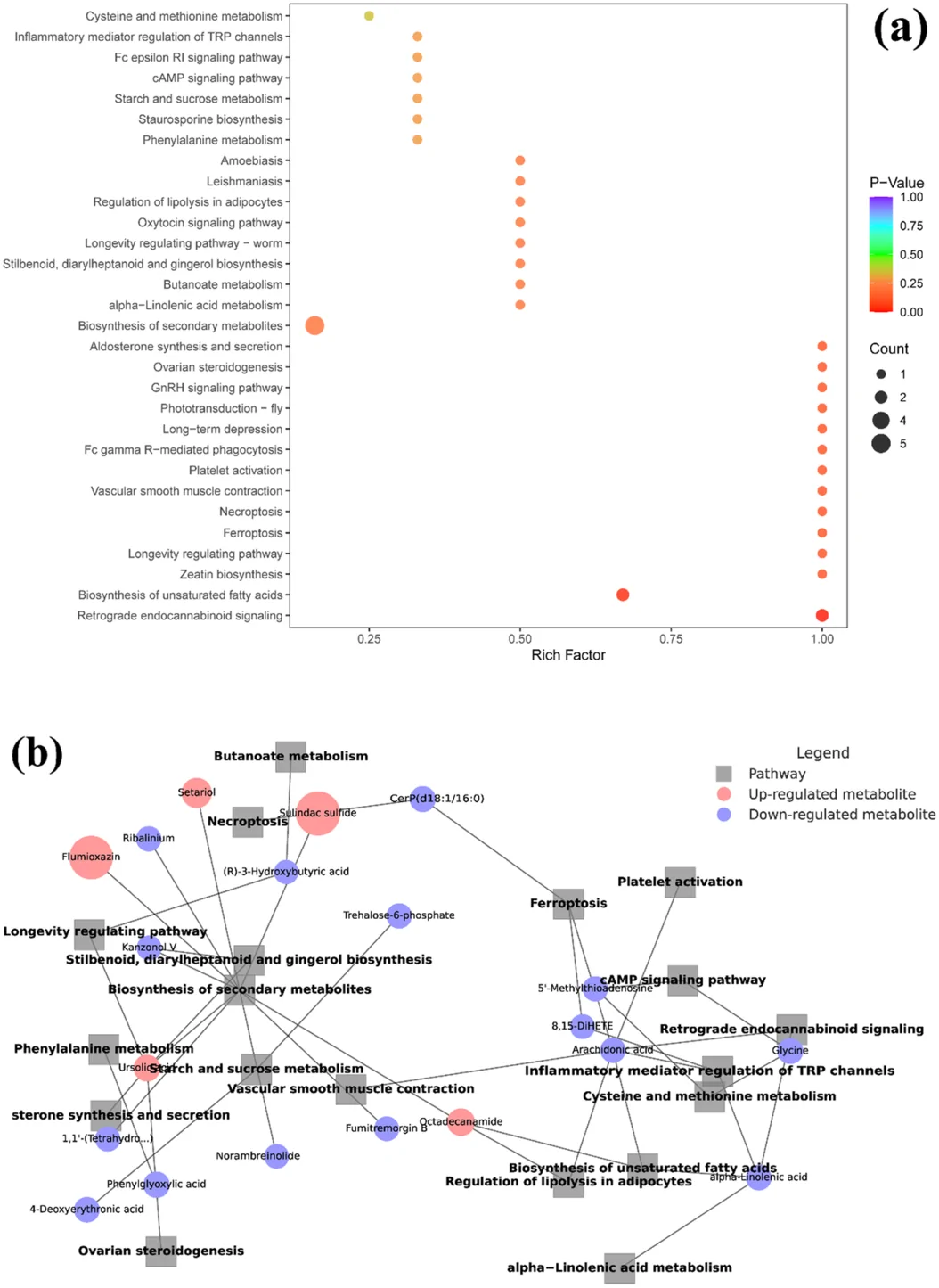
KEGG pathway enrichment analysis of differential metabolites in the FT‐vs‐ST comparison group. (a) KEGG enrichment analysis of differential metabolic pathways, (b) pathway mapping of soil differential metabolites. In panel (a), the closer the dot color is to red, the higher the significance of the difference. In panel (b), gray squares represent differential pathways, pink spheres represent upregulated differential metabolites and purple spheres represent downregulated differential metabolites, the larger the sphere, the higher the relative abundance. ST: soil with soybean and tetracycline; FT: soil with soybean, *F. mossea*e, and tetracycline.

Based on the differential metabolites identified in the FT‐vs‐ST treatment group, KEGG pathway enrichment analysis was performed (Figure 3a). The Rich factor represents the ratio of the number of differential metabolites enriched in a given pathway to the total number of metabolites annotated in that pathway, with a higher value indicating a greater degree of enrichment. The *p*‐value was used to evaluate statistical significance, and a lower value indicates more significant enrichment. A total of more than 30 differential metabolic pathways were identified after enrichment of all differential metabolites. Subsequently, according to the statistical significance results from MetaboAnalyst, 20 differential metabolites were mapped to the corresponding metabolic pathways, and 19 related pathways were obtained (Figure 3B), including retrograde endocannabinoid signaling, biosynthesis of unsaturated fatty acids, longevity regulating pathway, ferroptosis, necroptosis, vascular smooth muscle contraction, platelet activation, ovarian steroidogenesis, aldosterone synthesis and secretion, biosynthesis of secondary metabolites, α‐inolenic acid metabolism, butanoate metabolism, stilbenoid, diarylheptanoid and gingerol biosynthesis, Regulation of lipolysis in adipocytes, phenylalanine metabolism, starch and sucrose metabolism, cAMP signaling pathway, inflammatory mediator regulation of TRP channels, and cysteine and methionine metabolism. Using fold change and *p*‐value, we constructed a mapping network between differential metabolites and metabolic pathways with Python visualization tools (Figure 3b). The results showed that the polyunsaturated fatty acid and oxidative stress‐related cluster (e.g., α‐linolenic acid, arachidonic acid, 8,15‐DiHETE, and octadecanamide) was associated with biosynthesis of unsaturated fatty acids, α‐linolenic acid metabolism, ferroptosis, and inflammatory mediator regulation of TRP channels; the carbon source and energy metabolism cluster (e.g., (R)‐3‐hydroxybutyric acid, trehalose‐6‐phosphate, and 4‐deoxyerythronic acid) was involved in butanoate metabolism, Starch and sucrose metabolism, and longevity regulating pathway; the amino acid and sulfur‐containing metabolism cluster (e.g., glycine, 5'‐methylthioadenosine, and phenylglyoxylic acid) was linked to cysteine and methionine metabolism, phenylalanine metabolism, and cAMP signaling pathway; while the secondary metabolism and defense response cluster (e.g., ursolic acid, setariol, kanzonol V, fumitremorgin B, sulindac sulfide, and flumioxazin) was mainly involved in biosynthesis of secondary metabolites and stilbenoid/gingerol biosynthesis. Collectively, these results indicate that colonization by *F. mosseae* significantly affected key metabolic pathways related to energy metabolism, amino acid and sulfur‐containing metabolism and secondary metabolism.

### 3.4 Correlation Analysis Between Soybean Rhizosphere Soil Metabolites and Microbial Relative Abundance

**Figure 4:**
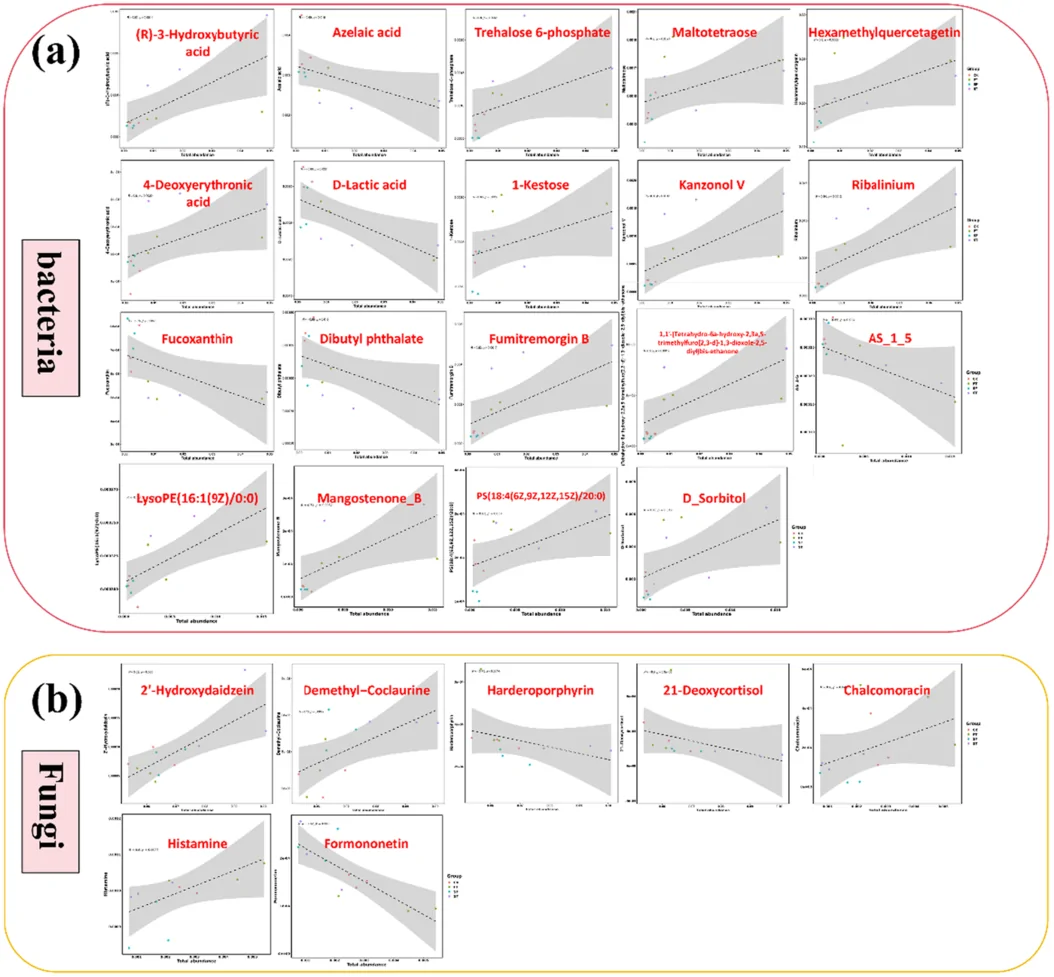
Correlation analysis between the summed relative abundance of differential microbial genera and differential metabolites in the soybean rhizosphere. (a) Correlation analysis between the summed relative abundance of differential bacterial genera and differential metabolites. (b) Correlation analysis between the summed relative abundance of differential fungal genera and differential metabolites. The x‐axis represents the summed relative abundance of differential bacterial or fungal genera in each biological sample and the y‐axis represents the relative abundance (peak area) of the differential metabolites. Each dot represents one biological replicate and different colors indicate different treatments. CK: soil only with soybean; SF: soil with soybean and *F. mosseae*; ST: soil with soybean and tetracycline; FT: soil with soybean, *F. mosseae*, and tetracycline.

Correlation analysis was performed between the summed relative abundance of differential bacterial or fungal genera and differential metabolites. It identified 19 metabolites that were significantly associated with bacterial relative abundance and 7 metabolites that were significantly associated with fungal relative abundance (Figure 4). Metabolites whose levels increased with rising bacterial relative abundance included (R)‐3‐hydroxybutyric acid (r = 0.81, *p* = 0.0024), trehalose‐6‐phosphate (r = 0.77, *p* = 0.0053), maltotetraose (r = 0.74, *p* = 0.0082), hexamethylquercetagetin (r = 0.8, *p* = 0.0027), 4‐deoxyerythronic acid (r = 0.78, *p* = 0.0041), 1‐kestose (r = 0.66, *p* = 0.022), kanzonol V (r = 0.81, *p* = 0.0024), ribalinium (r = 0.81, *p* = 0.0024), fumitremorgin B (r = 0.8, *p* = 0.0032), 1,1'‐(tetrahydro‐6a‐hydroxy‐2,3a,5‐trimethylfuro[2,3‐d]‐1,3‐dioxole‐2,5‐diyl)bis‐ethanone (r = 0.81, *p* = 0.0024), lysoPE(16:1(9Z)/0:0) (r = 0.73, *p* = 0.01), mangostenone_B (r = 0.74, *p* = 0.0082), PS(18:4(6Z,9Z,12Z,15Z)/20:0) (r = 0.62, *p* = 0.037), and d‐sorbitol (r = 0.68, *p* = 0.019), whereas metabolites that decreased with increasing bacterial abundance included azelaic acid (r = −0.69, *p* = 0.017), d‐lactic acid (r = −0.68, *p* = 0.019), fucoxanthin (r = −0.78, *p* = 0.0041), dibutyl phthalate (r = −0.68, *p* = 0.019), and AS 1‐5 (r = −0.66, *p* = 0.024) (Figure 4A). Metabolites whose levels increased with rising fungal relative abundance included 2'‐hydroxydaidzein (r = 0.62, *p* = 0.035), demethylcoclaurine (r = 0.81, *p* = 0.0024), chalcomoracin (r = 0.6, *p* = 0.043), and histamine (r = 0.8, *p* = 0.0027), whereas metabolites that decreased included harderoporphyrin (r = −0.75, *p* = 0.0074), 21‐deoxycortisol (r = −0.9, p = 5.9 × 10^−6^), and formononetin (r = −0.82, *p* = 0.002) (Figure 4B). Notably, 1,1'‐(tetrahydro‐6a‐hydroxy‐2,3a,5‐trimethylfuro[2,3‐d]‐1,3‐dioxole‐2,5‐diyl)bis‐ethanone, 4‐deoxyerythronic acid, kanzonol V, ribalinium, (R)‐3‐hydroxybutyric acid, and trehalose‐6‐phosphate all significantly increased with bacterial relative abundance but showed relatively weak correlations with fungi. Overall, correlations between rhizosphere soil metabolites and bacteria were stronger than those with fungi.

### 3.5 Analysis of the Relationships Between Soil Differential Metabolites and Individual Microorganisms

**Figure 5:**
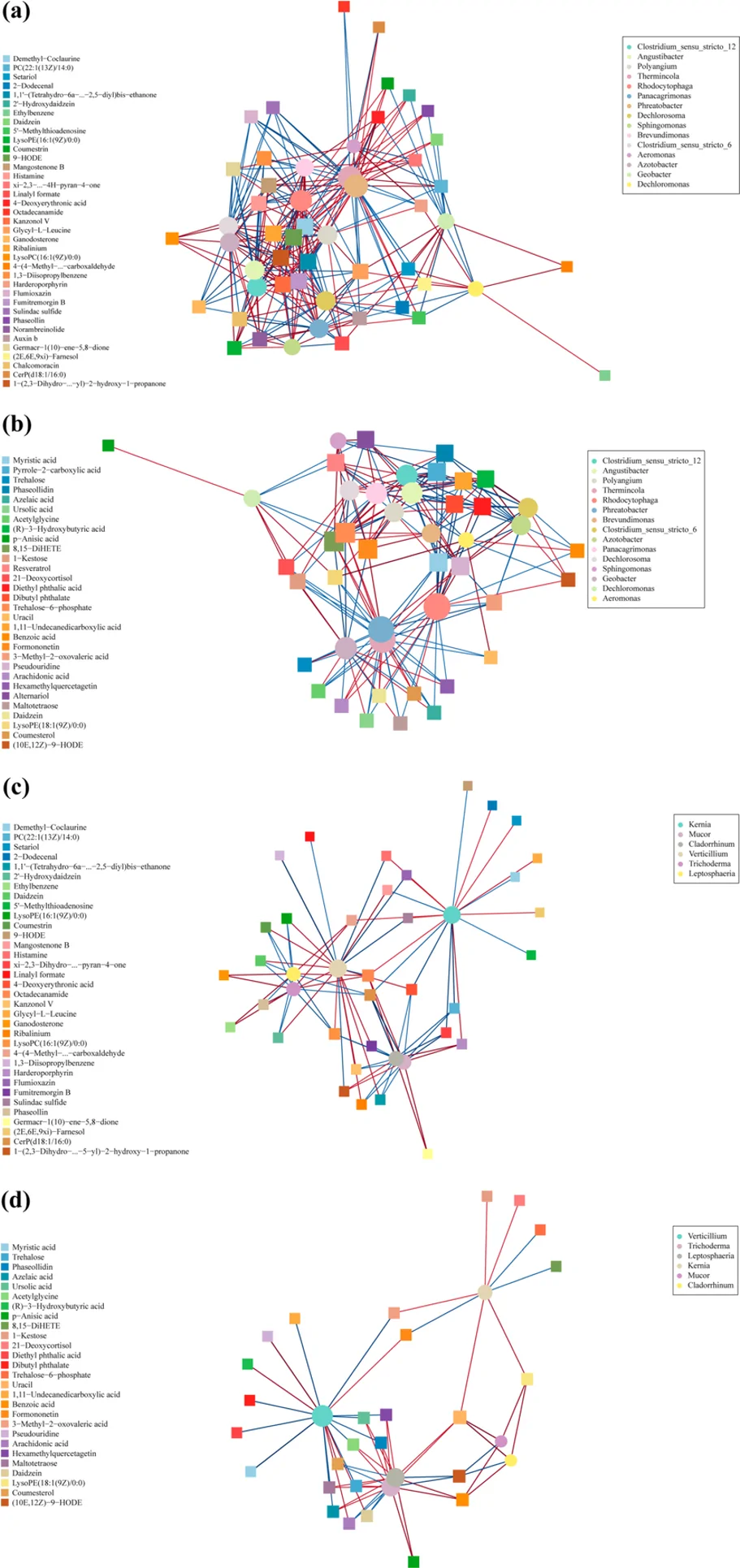
Correlation network between rhizosphere soil microbial genera and differential metabolites in soybean. (a) Correlation analysis between soil bacteria and positive ion metabolites, (b) correlation analysis between soil bacteria and negative ion metabolites, (c) correlation analysis between soil fungi and positive ion metabolites, (d) correlation analysis between soil fungi and negative ion metabolites. In the figure, different colored squares represent different metabolites, and different circles represent distinct microbial taxa. Connecting lines in warm colors indicate positive correlations, while connecting lines in cool colors indicate negative correlations. ST: soil with soybean and tetracycline; FT: soil with soybean, *F. mosseae* and tetracycline.

In the FT‐vs‐ST comparison, 16 differential metabolites were significantly correlated with 8 bacterial genera (Figure 5a–b). Among them, *Azotobacter* exhibited the strongest positive correlation with (R)‐3‐hydroxybutyric acid (r = 1.00, *p* = 0.0028) and was also positively correlated with kanzonol V, ribalinium, and fumitremorgin B (r > 0.94). Similarly, *Sphingomonas* were positively correlated with norambreinolide, 8,15‐DiHETE, and trehalose‐6‐phosphate (r ≈ 0.94), while *Phreatobacter* showed strong positive correlations with 4‐deoxyerythronic acid (r = 0.99, *p* < 0.001), trehalose‐6‐phosphate, and arachidonic acid. In contrast, *Dechlorosoma* exhibited significant negative correlations with 8,15‐DiHETE and trehalose‐6‐phosphate (r = −0.99, *p* < 0.001) and *Polyangium* was consistently negatively correlated with kanzonol V, ribalinium, and fumitremorgin B. A similar pattern was observed in fungi, where 12 differential metabolites were significantly correlated with 6 fungal genera (Figure 5c–d). *Verticillium* displayed positive correlations with (R)‐3‐hydroxybutyric acid (r = 0.94, *p* = 0.017), 4‐deoxyerythronic acid, and kanzonol V, but negative correlations with Flumioxazin and sulindac sulfide (r ≈ −0.94, *p* < 0.05). *Cladorrhinum* showed strong negative correlations with CerP(d18:1/16:0) (r = −1.00, *p* = 0.0028), ribalinium, and fumitremorgin B, while *Trichoderma* and *Leptosphaeria* were positively correlated with octadecanamide and ursolic acid but negatively correlated with arachidonic acid. Notably, stress‐related metabolites such as trehalose‐6‐phosphate and arachidonic acid exhibited opposite associations among different microbial groups: they were positively correlated with *Phreatobacter*, *Sphingomonas*, and *Verticillium*, but negatively correlated with *Dechlorosoma*, *Geobacter*, *Cladorrhinum*, and *Trichoderma*. This indicates that bacterial and fungal taxa possess functional differences in regulating stress‐responsive metabolic pathways. Overall, beneficial microbes such as *Azotobacter*, *Phreatobacter*, *Sphingomonas* and *Verticillium* were positively associated with the accumulation of protective or bioactive metabolites, whereas *Dechlorosoma*, *Polyangium*, *Cladorrhinum* and *Geobacter* were negatively associated with metabolites. These findings highlight the complementary yet antagonistic roles of bacteria and fungi in shaping rhizosphere metabolic responses under antibiotic stress. These results suggest that *F. mosseae* colonization was associated with simultaneous changes in microbial community composition and soil metabolite profiles.

## 4 Discussion

Soil metabolites primarily originate from plant root exudates and microbial metabolic activities. The composition of root exudates is strongly influenced by plant species, genotype, and environmental stresses (Duret et al. 2024; Luo et al. 2015). Our results demonstrated that tetracycline stress and *F. mosseae* colonization significantly altered the composition and abundance of rhizosphere soil metabolites, indicating that AMF symbiosis plays an important role in regulating rhizosphere metabolic responses under antibiotic stress.

### 4.1 *F. mosseae* Alters the Accumulation of Soil Metabolites Under Tetracycline Stress

Changes in soil metabolites often reflect the responses of plants and microorganisms to environmental stress. Antibiotic stress is known to disrupt metabolic pathways associated with lipid oxidation, inflammation regulation and cellular signal transduction. Our results (Figure 2) showed that under tetracycline stress, colonization by *F. mosseae* significantly altered the levels of several metabolites related to anti‐inflammatory and cytoprotective functions, suggesting that AMF colonization is associated with metabolic adjustments in the host–microbiome system under tetracycline stress. Firstly, *F. mosseae* colonization significantly increased the level of sulindac sulfide. Sulindac sulfide has been reported to possess anti‐inflammatory activity by inhibiting cyclooxygenase‐mediated conversion of arachidonic acid into pro‐inflammatory prostaglandins (Chakraborty et al. 2014). In the present study, the increased abundance of sulindac sulfide, together with the reduced level of its related metabolite 8,15‐DiHETE, may reflect metabolic reprogramming associated with AMF colonization under tetracycline stress. However, because these metabolites were identified using an untargeted metabolomics approach, their biological roles in the AMF–soybean symbiotic system should be interpreted with caution and require further validation. In addition, our study found that the content of ursolic acid continuously increased in the presence of *F. mosseae*. Ursolic acid is a pentacyclic triterpenoid with antioxidant, anti‐inflammatory and antimicrobial properties and can alleviate glutamate accumulation–induced oxidative damage and inflammation by enhancing neuronal function (Al‐Nasser et al. 2022; Kashyap et al. 2016). Its continuous accumulation may indicate that AMF colonization is associated with enhanced metabolic adaptation of the rhizosphere to tetracycline stress through the regulation of secondary metabolism.

Under tetracycline stress, the colonization of *F. mosseae* markedly altered the metabolic profile of the soybean rhizosphere, characterized by the reduced accumulation of multiple classes of metabolites, reflecting a systemic reprogramming of carbon–nitrogen allocation and microbial functional traits. We observed a decrease in the levels of glycine and trehalose‐6‐phosphate (T6P), which may result from AMF‐mediated uptake and translocation of free amino acids in soil as well as changes in host carbon–nitrogen partitioning, thereby reducing glycine in the rhizosphere (Whiteside et al. 2012). Meanwhile, AMF colonization and shifts in the microbial community could influence trehalose synthesis and degradation pathways, leading to a reduction in trehalose‐6‐phosphate levels (Kaur et al. 2022). In addition, tetracycline itself can disrupt rhizosphere microbial activity and nitrogen fixation, further exacerbating the reprogramming of amino acid and sugar metabolism (Sharma et al. 2020; Zhang et al. 2024). Therefore, the declines in glycine and trehalose‐6‐phosphate are likely the combined result of AMF metabolic regulation and antibiotic stress. Previous reports indicated that alpha‐linolenic acid functions as a signaling molecule under stress conditions and serves as the precursor of plant oxylipins and jasmonic acid (Sood 2023); it enhances stress tolerance by modulating membrane fluidity, acts as a regulator for plant defense gene activation, and promotes fungal spore growth and colonization (Yuan et al. 2022). Thus, the decreased level of alpha‐linolenic acid may be due to increased uptake driven by *F. mosseae* colonization and the stress‐induced demand from both plants and microorganisms. CerP(d18:1/16:0), an important signaling lipid associated with membrane homeostasis and stress responses, was also reduced, suggesting inhibition or redistribution of lipid signaling pathways in the rhizosphere (Zeng et al. 2022). Similarly, the decline in the aromatic intermediate phenylglyoxylic acid indicates that aromatic amino acid and phenylpropanoid metabolism is being rewired to synthesize other defense‐related compounds or further metabolized (Ninkuu et al. 2025). In sulfur metabolism, the decrease in 5′‐methylthioadenosine (MTA) implies disruption in the methionine cycle and polyamine metabolism (Tang et al. 2022). Meanwhile, the reduction in (R)‐3‐hydroxybutyric acid, a carbon storage‐related metabolite, reflects limitations in microbial fermentation and energy storage pathways (Prudnikova et al. 2024).

Other metabolites also indicated a decline in various plant or fungal‐derived secondary compounds. For example, norambreinolide and kanzonol V, sesquiterpene and benzofuran derivatives with defensive or signaling functions, were reduced, potentially reflecting a redistribution of metabolism between AMF, the host plant, and fungal communities (Toppo et al. 2023; van Dinteren et al. 2025). The decrease in the fungal‐derived toxin fumitremorgin B further suggests that the abundance or activity of toxin‐producing fungi was suppressed by the combined effects of AMF colonization and tetracycline stress (Zhou et al. 2021). In addition, the reduction of lipid signaling molecules such as arachidonic acid indicates weakened membrane lipid oxidation signaling and oxylipin‐based defense responses (Yang et al. 2025), while the decline of sugar acid intermediates such as 4‐deoxyerythronic acid suggests that carbon flow may be redirected to support AMF hyphal growth and host repair metabolism (Brtnicky et al. 2025). Some characteristic small molecules, including ribalinium and the complex structure 1,1'‐(tetrahydro‐6a‐hydroxy‐2,3a,5‐trimethylfuro[2,3‐d]‐1,3‐dioxole‐2,5‐diyl)bis‐ethanone, also showed downward trends, although these compounds are rarely reported and remain poorly understood. Overall, the systemic decrease in these metabolites revealed that AMF colonization, under tetracycline stress, reshapes the rhizosphere metabolic environment through carbon–nitrogen reallocation, microbial community restructuring, and downregulation of secondary metabolism, thereby providing a metabolic basis for mitigating antibiotic pressure and maintaining symbiotic mutualism in the host plant.

### 4.2 The Effects of *F. mosseae* on Soil Microbial Metabolic Pathways Under Tetracycline Stress

In this study, KEGG enrichment analysis of differential metabolites indicated that the inoculation of *F. mosseae* significantly influenced multiple metabolic pathways, including lipid oxidation, carbon energy metabolism, amino acid and sulfur metabolism, as well as secondary metabolism (Figure 3). Overall, the present study supported both hypotheses proposed in the introduction. First, *F. mosseae* significantly reshaped the composition of rhizosphere soil metabolites and altered microbial metabolic pathways under tetracycline stress. Second, differential metabolites showed significant associations with specific bacterial and fungal taxa, indicating that changes in metabolite profiles were closely linked to microbial activity and community structure. Together, these findings provide new mechanistic insights into how AMF regulates rhizosphere microbial metabolism and contributes to the mitigation of antibiotic stress in agricultural soils.

Firstly, regarding lipid oxidation, polyunsaturated fatty acids (PUFAs) such as α‐linolenic acid, arachidonic acid, 8,15‐DiHETE and octadecanamide were predominantly enriched in the ferroptosis pathway, suggesting a potential mechanism in the soil microbial symbiotic system for modulating oxidative stress through lipid peroxidation. This is consistent with recent studies on ferroptosis, which indicate that the integration and oxidation of PUFA‐containing membrane lipids serve as key triggers for ferroptosis (Kim et al. 2023). Therefore, it can be hypothesized that *F. mosseae* may reprogram the ferroptosis metabolic pathway in rhizosphere microbes by regulating host plant signaling, thereby enhancing oxidative stress tolerance. In some plant studies, PUFAs are also implicated in antioxidant defense rather than necessarily promoting lipid peroxidation (Suda et al. 2024), highlighting species‐specific differences in PUFA functions.

Secondly, in terms of carbon and energy metabolism, (R)‐3‐hydroxybutyric acid, trehalose‐6‐phosphate, and 4‐deoxyerythronic acid were enriched in pathways such as butanoate metabolism, starch and sucrose metabolism, and the longevity regulating pathway. This suggests that the presence of *F. mosseae* induces a reprogramming of soil microbial carbon metabolism, maintaining carbon allocation and energy homeostasis within the symbiotic system. These findings align with those of Liu et al. (2024), who reported that AM symbiosis significantly increased the contribution of plant‐derived carbon to soil metabolites and altered KEGG pathways associated with carbohydrate metabolism. Similarly, Ma et al. (2025) demonstrated that AMF plays a crucial role in regulating the contributions of plants and soil microbes to soil organic carbon. Comparable phenomena have been reported in plant–fungus interactions, where AMF symbiosis promotes host sugar metabolism and organic acid secretion to support fungal colonization (De Rose et al. 2023). Notably, the significant enrichment of the Longevity regulating pathway is rarely observed in symbiotic systems, suggesting that its associated signaling may be reactivated under AMF symbiosis and warrants further investigation.

Thirdly, regarding amino acid and sulfur metabolism, glycine, 5′‐methylthioadenosine, and phenylglyoxylic acid were enriched in cysteine and methionine metabolism, phenylalanine metabolism, and the cAMP signaling pathway. Sulfur‐containing amino acids serve both as building blocks for protein synthesis and as precursors for antioxidant molecules such as glutathione, playing critical roles in maintaining redox homeostasis. Previous studies have reported that AMF enhance the enrichment of genes involved in amino acid synthesis, aminoacyl‐tRNA biosynthesis, and cysteine and methionine metabolism (Li et al. 2024). In plant–fungi interaction systems, sulfur metabolism in both plants and microbes is vital for nutrient exchange and stress responses (De Rose et al. 2023). Additionally, fungal amino acid metabolism is decisive for hyphal growth and symbiotic adaptability (Ni et al. 2024). Our findings are consistent with these reports, suggesting that amino acid and sulfur metabolism in the *F. mosseae*–soybean symbiotic system may simultaneously support nutrient interactions between microbes and plants and participate in signaling regulation.

Finally, in terms of secondary metabolism, metabolites such as ursolic acid, kanzonol V, fumitremorgin B, and sulindac sulfide were mapped to the biosynthesis of secondary metabolites and stilbenoid/gingerol biosynthesis pathways. These compounds are generally associated with defense responses and antioxidant functions, indicating that *F. mosseae* colonization may induce the synthesis of microbial secondary metabolites in the rhizosphere to modulate defense signaling or counteract environmental stress. Similarly, reviews have emphasized the central role of secondary metabolites in fungal–plant interactions for signaling and defense responses (Elhamouly et al. 2022). Unlike some studies reporting that AMF downregulate certain defensive metabolites to minimize disruption of the symbiotic relationship, we observed upregulation in these secondary metabolic pathways, which may reflect differences in microbial composition or metabolic strategies under antibiotic stress.

In summary, our results suggested that under antibiotic stress, AMF reprogrammed multiple metabolic pathways, collaboratively regulating lipid oxidation, carbon energy metabolism, amino acid and sulfur metabolism, and secondary metabolism networks. This may enhance the metabolic homeostasis and stress resilience of soil microbes. This metabolic pattern aligns with previously reported symbiotic regulatory mechanisms, while also highlighting distinct responses under specific environmental stress conditions, providing new evidence for understanding how AMF–plant interactions modulate rhizosphere microbial metabolism.

### 4.3 Colonization of *F. mosseae* Alters the Links Between Various Metabolites and Microbes Under Tetracycline Stress

Soil microorganisms are key executors of soil metabolic activities, and their detectable biological responses are directly reflected in changes in microbial composition and abundance under various conditions. More importantly, changes in microbial composition and abundance partly determine the dynamics of soil metabolites, which in turn influence the cycling and utilization of exogenous nutrients in the soil. Therefore, uncovering the correlations between soil metabolites and microbial communities is of great significance (Cheng et al. 2022). Previous studies have shown that metabolites are significantly correlated with endophytic fungi (Cui et al. 2019), and Song et al. (2020) also reported positive or negative correlations between soil metabolites and the relative abundance of differential bacteria. In this study, changes in soil metabolite levels were significantly correlated with the relative abundance of both bacteria and fungi, further supporting the idea that soil microorganisms can promote or suppress the synthesis of secondary metabolites. In the monomer correlation network analysis, both soil bacteria and fungi exhibited significant interactions with differential metabolites (Figure 5). Among bacteria, *Azotobacter* showed strong positive correlations with (R)‐3‐hydroxybutyric acid and multiple secondary metabolites (e.g., kanzonol V, fumitremorgin B), suggesting that this genus may play a key role in soil ecosystems by promoting carbon conversion and metabolite accumulation (Nagajothi and Murugesan 2023). *Sphingomonas* was correlated with trehalose‐6‐phosphate and 8,15‐DiHETE, indicating its potential involvement in regulating antioxidant and stress‐related metabolism in soil (Asaf et al. 2017). In contrast, *Dechlorosoma* showed significant negative correlations with trehalose‐6‐phosphate and arachidonic acid (Su et al. 2018), which may imply the suppression of certain protective metabolic processes under stress conditions.

The fungal community also displayed differentiation (Figure 5c,d). For example, *Verticillium* was positively correlated with (R)‐3‐hydroxybutyric acid and kanzonol V, suggesting that it may be closely associated with organic matter transformation and active metabolite accumulation in the soil metabolic network (Su et al. 2018). In contrast, *Cladorrhinum* showed negative correlations with multiple metabolites, including CerP(d18:1/16:0) and fumitremorgin B, potentially weakening the accumulation of specific compounds through competitive or inhibitory mechanisms (Barrera et al. 2019). Furthermore, typical stress‐related metabolites such as trehalose‐6‐phosphate and arachidonic acid exhibited opposite correlation directions across different microbial groups, indicating that bacteria and fungi play both complementary and antagonistic roles in regulating soil metabolic pathways.

It should be noted that the correlations observed in the present study do not necessarily imply direct causal relationships between microbial taxa and differential metabolites. A more plausible explanation is that *F. mosseae* colonization first alters plant root exudation and rhizosphere metabolite profiles, which subsequently influence the recruitment and assembly of specific microbial taxa. In turn, the reshaped microbial community may further modify rhizosphere metabolism through microbial transformation and nutrient cycling. Therefore, the observed metabolite–microbe associations are more likely to represent coordinated responses within the AMF–plant–soil system than direct cause‐and‐effect relationships. Overall, the present study supported both hypotheses proposed in the introduction. First, *F. mosseae* significantly reshaped the composition of rhizosphere soil metabolites and altered microbial metabolic pathways under tetracycline stress. Second, differential metabolites showed significant associations with specific bacterial and fungal taxa, indicating coordinated changes in metabolite profiles and microbial community composition under *F. mosseae* colonization. Together, these findings provide new mechanistic insights into the coordinated responses of rhizosphere metabolites and microbial communities to *F. mosseae* colonization under tetracycline stress.

## 5 Conclusion

This study demonstrated that *F. mosseae* reshaped the interactions among the soybean rhizosphere, soil microorganisms and their associated metabolites under tetracycline stress. A total of 34 differential metabolites identified in the FT‐vs‐ST comparison were mainly involved in primary metabolism, secondary metabolism and defense‐related processes. These metabolites were further enriched in 19 metabolic pathways, which could be classified into four major functional groups: polyunsaturated fatty acid and oxidative stress‐related metabolism, carbon and energy metabolism, amino acid and sulfur‐containing metabolism and secondary metabolism and defense responses. Furthermore, integrated analyses revealed significant associations between differential metabolites and specific bacterial and fungal taxa, indicating that *F. mosseae* regulates rhizosphere microbial metabolism through coordinated microbe–metabolite interactions. Collectively, these findings provide new insights into the metabolic mechanisms by which AMF alleviates tetracycline stress and improve our understanding of microbe–metabolite interactions in the soybean rhizosphere.

## Author Contributions

**Chao Yang:** writing – review and editing, conceptualization, data curation, investigation, formal analysis, writing – original draft. **Baiyan Cai:** conceptualization, project administration, funding acquisition, writing – review and editing, methodology. **Harry Lye Hin Chong:** writing – review and editing. **Juan Li:** investigation, data curation. **Linghui Li:** data curation, investigation. **Jean Wan Hong Yong:** funding acquisition, writing – review and editing, resources. **Khim Phin Chong:** conceptualization, supervision, writing – review and editing.

## Conflicts of Interest

The authors declare no conflicts of interest.

## Supporting information


Supporting File

## Data Availability

The data that supports the findings of this study are available in the supplementary material of this article. All data generated or analyzed during this study are included in this published article and its additional files.
