# Supplementary material for: The Mycorrhizal Fungal Species Funneliformis mosseae Reshapes Rhizosphere Microbial Metabolic Pathways of Soybeans Under Tetracycline Stress
Source: Microbiologyopen. 2026 Aug 30;15(5):e70385. doi: 10.1002/mbo3.70385 (PMC13527221; doi:10.1002/mbo3.70385)
Supplement: Supplementary file 1 [file MBO3-15-e70385-s001.docx]

Supplementary material

**The mycorrhizal fungal species *Funneliformis mosseae* reshapes rhizosphere microbial metabolic pathways of soybeans under tetracycline stress**

Chao Yang ^a,b^, Baiyan Cai ^c^, Harry Lye Hin Chong ^b^, Juan Li^a^, Linghui Li^a^, Jean Wan Hong Yong^d,e^*, Khim Phin Chong ^b^*

^a^ *Faculty of Science, Heihe University, Heihe 164300, Heilongjiang, China*

^b^ *Faculty of Science and Technology, Universiti Malaysia Sabah, Jalan UMS, Kota Kinabalu, 88400, Sabah, Malaysia.*

^c^ *Engineering Research Center of Agricultural Microbiology Technology, Ministry of Education & Heilongjiang Provincial Key Laboratory of Plant Genetic Engineering and Biological Fermentation Engineering for Cold Region & Key Laboratory of Microbiology, College of Heilongjiang Province & School of Life Sciences, Heilongjiang University, Harbin, 150080, China*

*^d^Department of Biosystems and Technology, Swedish University of Agricultural Sciences, Alnarp,, Sweden*

*^e^* *Brightlands Future of Farming Institute, Faculty of Science and Engineering, Maastricht University, Venlo, Netherlands*

* Corresponding author.

E-mail address: [jean.yong@slu.se](mailto:jean.yong@slu.se) (Jean Wan Hong Yong)

Department of Biosystems and Technology, Swedish University of Agricultural Sciences, Alnarp, 23456, Sweden

E-mail address: [chongkp@ums.edu.my](mailto:chongkp@ums.edu.my) (Khim Phin Chong)

Faculty of Science and Technology, Universiti Malaysia Sabah, Jalan UMS, Kota Kinabalu 88400, Sabah, Malaysia.

**Contents:**

3 figures and 3 tables.

**Table_S1. Soil condition before planting**

|  | pH | TOM | TN | TP | TK |
| --- | --- | --- | --- | --- | --- |
|  | - | g/kg | mg/kg | g/kg | g/kg |
| Before experiment | 7.36±0.03 | 29.51±0.35 | 1460.03±15.64 | 983.73±5.01 | 15.72±0.57 |
|  | AN | AP | AK | Clay content | σ |
|  | mg/kg | mg/kg | mg/kg | % | μS·cm^-1^ |
| Before experiment | 165.05±1.58 | 47.96±0.39 | 249.78±0.56 | 12.38±0.16 | 579.93±6.21 |

**TOM: Total Organic Matter, TN: Total Nitrogen, TP: Total Phosphorus, TK: Total Potassium, AN: Available Nitrogen, AP: Available Phosphorus, AK: Available Potassium, σ: Conductivity (elec.).**

**Table_S2. The fat and protein content in HN48 soybean**

| Class | Acronyms | Fat contents (%) | Protein content (g/100g) |
| --- | --- | --- | --- |
| Hei Nong 48 | HN48 | 18.43 | 45.23 |

**Table_S3. Experimental Design**

|  | Group | TC  （mg） | *F.mosseae*  (g) | Sterilized soil  (g) | Sterilized soil  (mg) | Soybean  (Plant) |
| --- | --- | --- | --- | --- | --- | --- |
| Control group | CK | - | - | 40 | 160 | 3 |
| Treatment group | ST | 160 | - | 40 | - | 3 |
|  | SF | - | 40 | - | 160 | 3 |
|  | FT | 160 | 40 | - | - | 3 |

**CK: soil only with soyabean; SF: soil with soyabean and *F. mosseae*; ST: soil with soyabean and tetracycline; FT: soil with soyabean and *F. mosseae* and tetracycline**

**Figure S1. Total ion chromatograms (TICs) of metabolites from all soil samples and QC samples analyzed by UPLC-MS. CK: soil only with soybean; SF: soil with soybean and *F. mosseae*; ST: soil with soybean and tetracycline; FT: soil with soybean and *F. mosseae* and tetracycline**

**Figure_S2. KEGG pathway annotation map for tetracycline-associated soil metabolites in the rhizosphere of *F. mosseae*-treated soybean**

**Figure S3. Hierarchical clustering heatmap analysis of differential metabolites in pairwise comparison groups. The upper panel shows positive ion mode, and the lower panel shows negative ion mode. The solid lines on the left side of the heatmap represent clustering analysis. CK: soil only with soybean; SF: soil with soybean and *F. mosseae*; ST: soil with soybean and tetracycline; FT: soil with soybean and *F. mosseae* and tetracycline**
